# Extemporised Hospitals

**Published:** 1914-08-15

**Authors:** 


### Emergency Work in the Provinces.

### EXTEMPORISED HOSPITALS.

Amongst the most important duties devolving on Voluntary Aid Detachments is the improvising of hospitals. This branch of their duties extends to the collection of sick and wounded by field ambulances, the receipt of such wounded at a Clearing hospital, and the removal when possible of these patients to a Temporary hospital. We may add that the Temporary hospital in the Territorial system holds an equivalent place to that of the Stationary hospital in the medical organisation of the Regular Forces. It is laid down that the Voluntary Aid Detachments should make a survey of every county, and then place on permanent record the capacity of every village and town for receiving sick and wounded, and for providing hospital accommodation for them. Maps should be prepared of each county, on which should be marked the accommodation at each village and township for sick and wounded, the existing water supply, the sanitary condition, any suggested alteration for provision of kitchens and in fulfilment of sanitary requirements, the surgeries of medical practitioners willing to assist, and the strength of the Voluntary Aid Detachments in each place throughout the district. It is essential that all this information should be collected and made available before the outbreak of war, so that the necessary arrangements for accommodating the sick and wounded, and passing them on from the field ambulance through Clearing and Temporary hospitals to the Base hospitals, can proceed in regular sequence without delay or difficulty. If the British Red Cross Society has done its work thoroughly, the initial organisation throughout the country should be remarkably good. **We should like to have accurate information on this point, and shall hope to receive communications from someone in charge of every Voluntary Aid Detachment, as it is our purpose to help this movement, and to record its work and usefulness week by week.**

### THE WORK OF THE VOLUNTARY AID DETACHMENTS.

The Voluntary Aid Detachments and the Red Cross are not required to work in the firing line. The surgeons and their orderlies will do all that is required in the firing line to save life and to stay hæmorrhage. The Voluntary Aid bearers have to await the advance of the firing line, when the field ambulances follow and collect the wounded where they may have been placed under shelter in different parts of the field. The field ambulance next takes the wounded back to the Clearing hospital, where they remain until passed on to the Temporary hospital and so forth as already explained. Sometimes the field ambulance has attached to it nursing sections, where tents are pitched and operating theatres and kitchens provided. Here the wounded can be thoroughly treated, and can remain for two or three days, or a few hours, as the nature of their cases may demand. The Voluntary Aid Detachments throughout the English counties have been exceedingly active during the past ten days. The inspecting officers appear to receive great satisfaction from the good work done through the Red Cross Society and its members. The chief need is that the whole of the women should close their ranks, and set to work with a will to make themselves more perfect by practice than they are at present.

Brighton.

The Royal Sussex County Hospital Committee has adopted the unique course, after fulfilling its agreement to supply a contingent of nurses for military service in the event of war, and in the absence of some of its surgeons and staff who have been summoned to the colours, to close some of its wards. This is a procedure which is quite unique so far as this country is concerned, seeing that all other hospital managers realise that shortly there may be infinitely greater pressure upon their beds owing to the demand due largely to the state of war on many poor persons. And all other voluntary hospitals have properly declined to permit their staffs of doctors and nurses to be depleted to any extent which would interfere with the efficient upkeep and working of the whole of the available beds at their disposal. A reference to page 553 will show that Brighton is apparently the only centre without a principal matron for its Territorial Hospital. It is a sad thing that one of the richest communities in the neighbourhood of London should show such a lack of administrative perception and energy. Fancy a plutocratic community closing hospital beds, even temporarily, at such a time as the present!

The Royal Naval Hospital, Southend-on-Sea.

Her Majesty Queen Mary has accepted the presidency of this institution, and an urgent appeal has been issued by the Duke and Duchess of Portland, Sir Alfred D. Fripp, Dr. William Hale White, and others for funds to support the hospital, which has been instituted with the object of allaying the sufferings of the sick and wounded of the Royal Navy. Owing to the patriotism and generosity of the executors of the late Mr. Alfred Tolhurst, the Palace Hotel, Southend-on-Sea, has been placed at the disposal of the committee free of rent during the continuance of the war. This building will accommodate at least 400 patients, and is said to be in every way extremely well suited for hospital purposes. The hospital has been personally approved and accepted on behalf of the Admiralty by Sir Arthur May. Donations may be sent to Messrs. Coutts and Co., 440 Strand, W.C., or to Lady Maud Wilbraham, hon. treasurer, 26 Lower Sloane Street, S.W., to the credit of Queen Mary's Royal Naval Hospital, Southend-on-Sea, account.

Nottingham.

The Duke and Duchess of Portland are the presidents of this branch, and they not only take a deep interest in the work, but are most liberal and hospitable to the workers. West Park, Welbeck, has been lent to the Detachments for their annual gatherings for some years, and recently 800 men and women belonging to the Notts Voluntary Aid Detachments were present. On this occasion an improvised hospital tent was erected, containing eight beds, an operating theatre, and the necessary equipment for field cooking, laundry work, etc. These adjuncts were provided to enable the War Office to hold an examination of three Detachments. Various manœuvres and operations were carried out to the great interest of the large number of people assembled, and the whole proceedings passed off satisfactorily, and reflected great credit upon all concerned.

Leeds.

The hospital preparations of Leeds include the Second Northern General Hospital Section of the Territorial Force, which is in charge of Major J. F. Dobson, R.A.M.C., Major J. A. Copland, and Lieut. Sedgwick. Forty members of this corps are at present completing the arrangements. The organisation for the equipment of the hospital includes twenty medical practitioners, mostly members of the Leeds Infirmary staff, and ninety-two nurses. An additional Voluntary Aid staff of sixty-six is held in reserve. To commence with, only 200 beds are provided, but arrangements have been made to increase the number to 500, and then, should occasion arise, to erect marquees in the grounds, which are amply sufficient to enable 2,000 beds to be so provided. The nursing arrangements are in charge of Miss Innes, of the General Infirmary, and her assistant will be Miss Hill, matron of the Halifax Royal Infirmary. The St. John's Ambulance Association have been holding meetings at the Leeds University in order to enlist recruits, both men and women, for their Voluntary Aid Detachments on service. Lord Harewood, Lord-Lieutenant of the West Riding, has placed nearly the whole of his residence, Harewood Park, at the disposal of the Government for use for hospital purposes. Lord Harewood has already provided 50 beds. The Lord Mayor of Leeds, Mr. E. A. Brotherton, has provided another 50 beds for the wounded at the Hall, Roundhay. The Leeds Training College in Beckett's Park is being rapidly converted into a military hospital. The college is declared to be admirably adapted for this purpose.

Manchester.

The organisation of the Territorial ambulance services in this city is upon an extensive scale. The Manchester School of Technology and the two secondary grade schools in Whitworth Street have been commandeered to be used as general hospitals for the reception of sick and wounded soldiers. They are being fitted up with 550 beds, 500 for the men and fifty for officers. The total bed accommodation at the disposal of the Second Western General Hospital, East Lancashire Division of the Territorial Force, can, if need be, be extended to 1,100. The staff for the hospital just referred to will for the present be twenty-one officers, 130 men, and sixty-eight nurses, with a nucleus staff of 253. Sixty-six male volunteers are required who have served in the Royal Army Medical Corps or otherwise had ambulance experience. Contracts have been signed with various Manchester firms to supply all the necessaries that will be required. The work has proceeded with commendable rapidity, owing to previous experiments made in the organisation of the British ambulance service. Further schools have been commandeered at Fallowfield, Whalley Range, Rusholme, Stretford, Old Trafford, and other places. The Royal Infirmary staff of medical men, whose services are available with the Second Western General Hospital, have been mobilised by the War Office, and all the war nurses have been informed that their services may be required at any moment. The British Red Cross Society and the Voluntary Aid Detachments are making the fullest preparations for the work of receiving, housing, and nursing the wounded soldiers. The general scheme for the East Lancashire Branch is being worked out by the executive meeting. Executive committees are urgently called for in many places around Manchester and the district.

### Newcastle-upon-Tyne.

In Newcastle the Royal Victoria Infirmary has placed 200 beds at the disposal of the military authorities. These beds are vacant and are being kept vacant. Moreover, the 1st Northern General Hospital, Territorial Force, has been mobilised, and the Armstrong College, which adjoins the Infirmary, has been fitted up with beds. At the present time in this building (the Armstrong College) there are 300 beds ready and, when the necessary sanitary alterations have been completed, the Territorial hospital will have 520 beds. The staff of the Territorial hospital is composed of members of the staff of the Newcastle Royal Infirmary, and although the Territorial Force has first claim upon their services, so far as their military duties will allow, they will carry out the civil work in this hospital.

At the same time a committee is sitting to make arrangements to supplement the civil beds either by taking over one of the public buildings in the town, or by erecting temporary accommodation on the Moor, adjacent to and connected with this hospital. Numerous offers of private houses in Newcastle and district have been made, and the committee may be able to utilise these offers for convalescent cases.

Other institutions in the neighbourhood have, it is understood, placed beds at the disposal of the Admiralty and the military authorities. The Red Cross Society are also making provision for beds. At Alnwick, for instance, it is officially known that they have already equipped a hospital and can make provision for 400 beds; 100 are already available.

In Newcastle itself there is adequate provision for any wounded, naval or military, that may be brought in, and the hospital authorities are making every effort to secure that adequate provision should be made for the civil population.

### Sheffield.

The Third Northern General Hospital scheme under Lord Haldane's scheme through the War Office is to have Sheffield, Leeds, and Newcastle as the three northern cities which will serve as hospital centres in charge of the Royal Army Medical Corps. The buildings occupied by the Women's Hostel in Collegiate Crescent, the Men's Training College, and the Men's Hostel in Clark House Road have been transformed into a hospital base under Dr. Arthur Connell, the officer in command. From the earliest inception of the Third Northern General Hospital Sheffield has shown an unqualified approval of, and interest in, the scheme. Sheffield was the first city in the country where the Voluntary Aid Detachment reached its establishment strength. The men have only recently returned from their training at the York Military Hospital and are full of enthusiasm. Sheffield is the headquarters of the Field Ambulance Brigade of the R.A.M.C., which has made Red Cross wagons very familiar to the citizens.

